# Something Needed for the Worthy Doctor to Be Appreciated*Read before the Central Texas Medical Association, January 10, 1900, at Waco, Texas.

**Published:** 1900-03

**Authors:** R. H. Simpson

**Affiliations:** Turnersville, Texas


For Texas Medical Journal.

### Something Needed For the Worthy Doctor to Be Appreciated.*

BY R. H. SIMPSON, M. D., TURNERSVILLE, TEXAS.

GENTLEMEN:—You well know the attributes of a worthy doctor. But do the people at large realize that a worthy doctor must be of good moral character, must have a good primary education, and be a graduate of a medical college in harmony with the American Medical Association, and be a working member of the medical association where he lives? And do they know the real work of medical colleges and associations of today, and the labor of the worthy doctor outside of his regular routine work? Surely they do not know these things, therefore cannot properly appreciate the worthy doctor, and will never do so under our present code of medical ethics in central Texas.

Gentlemen, I take the position that the worthy doctor is to blame.

---

*Read before the Central Texas Medical Association, January 10, 1900, at Waco, Texas.

I cannot say, like the charlatan specialists in their private glee over victory, the doctors are acting Rip Van Winkle, for as a rule they are burning the midnight oil and drifting to early graves in poverty.

Gentlemen, we are betwixt the devil and the deep blue sea. The battle must be fought, and I say hoist the flag, cut loose the capstan, fire the signal gun, and launch out into the mighty deep. If our ship is worthy of a captain surely he will arm his men, and command them to batter down our enemies, which are pretended specialists, quacks, tender toed regulars, and last but not least, want of knowledge on the part of the laity.

Gentlemen, are you appreciated and remunerated? How is it the cold, dark night when the mother's anxiety reaches out for her suffering child? Then how is it when the bill matures?—Quite a different problem to solve. How is it when the pretended specialist comes into your midst? Do they not promise great things to those whom you have advised honestly, and take out large sums of money to which you are entitled? How is it when the unprofessional "Doc." locates in your midst, cuts prices and puts up the old song and dance to your people that you have been bleeding them?

Gentlemen, can we overcome these evils under our present system of business in a single handed way? Have we not gone to our legislature in behalf of humanity and battled with this state of affairs in vain?

Gentlemen, if a national bank wished to elect directors, would they elect a man who has been a financial failure? Or if a high school wished to elect directors, would they elect illiterate men? Most assuredly they would not. Then why should the worthy doctor appeal to our representatives for help and to regulate the practice of medicine so long as they are ignorant of the needs of the worthy doctor or prompted by some political craze or ambition?

Gentlemen, our enemies are entrenched in ignorance and firing on us as we attempt to march forward. Gentlemen, we must fall upon some plan by which to enlighten the laity. This association has done a great deal for the worthy doctor and to enlighten the people. I bid it God speed.

Gentlemen, I believe we need more thorough work, and our proceedings cast before the eyes of every man, woman and child in the bounds of this association. I also recommend the organization of subordinate medical associations wherever half a dozen regular physicians can be together quarterly, and let them discuss every question affecting them in any way in that locality, print their work, and place a copy in the hands of every individual in that locality. I

also recommend that each organization select a suitable man to publicly discuss anything that will enlighten the laity, and place the worthy doctor squarely upon his merits.

Gentlemen, I know that organizations are by no means like the pope, infallible. They are like individuals, subject to septic influences and imperfections. But in a multitude of counsel there is safety. Some will say that such a plan will cause dissentions, bickerings and wranglings.

I am sure no one would entertain for a moment the idea or hope of Dean Swift's commandment when introduced before the Lord Bishop of England, when he paraphrazed the subject handed him by saying "Love ye one another." For the success of an individual or an association never depends upon the peace and quiet of an uneventful career. It is the turmoils, the school of contention, the contact of mind versus mind that give individuality and prominence. History furnishes us many examples of contentions, disagreements, and even quarrels, that terminated far more amicably than do some of the honeymoons of the present day.

Gentlemen, I would not attempt to lead this body into a new departure, for of you all I feel myself the least, but submit the subject for your consideration. Should my plan meet with your approval, I suggest that this body take steps to put it into effect. Or draft some better plan by which the worthy doctor may become more appreciated and better known by the laity.

